# The effects of elevated seawater temperatures on Caribbean gorgonian corals and their algal symbionts, *Symbiodinium* spp.

**DOI:** 10.1371/journal.pone.0171032

**Published:** 2017-02-02

**Authors:** Tamar L. Goulet, Kartick P. Shirur, Blake D. Ramsby, Roberto Iglesias-Prieto

**Affiliations:** 1 Department of Biology, University of Mississippi, University, Mississippi, United States of America; 2 Unidad Académica de Sistemas Arrecifales, Instituto de Ciencias del Mar y Limnología, Universidad Nacional Autónoma de México, Puerto Morelos, Quintana Roo, Mexico; Academia Sinica, TAIWAN

## Abstract

Global climate change not only leads to elevated seawater temperatures but also to episodic anomalously high or low temperatures lasting for several hours to days. Scleractinian corals are detrimentally affected by thermal fluctuations, which often lead to an uncoupling of their mutualism with *Symbiodinium* spp. (coral bleaching) and potentially coral death. Consequently, on many Caribbean reefs scleractinian coral cover has plummeted. Conversely, gorgonian corals persist, with their abundance even increasing. How gorgonians react to thermal anomalies has been investigated utilizing limited parameters of either the gorgonian, *Symbiodinium* or the combined symbiosis (holobiont). We employed a holistic approach to examine the effect of an experimental five-day elevated temperature episode on parameters of the host, symbiont, and the holobiont in *Eunicea tourneforti*, *E*. *flexuosa* and *Pseudoplexaura porosa*. These gorgonian corals reacted and coped with 32°C seawater temperatures. Neither *Symbiodinium* genotypes nor densities differed between the ambient 29.5°C and 32°C. Chlorophyll *a* and *c*_*2*_ per *Symbiodinium* cell, however, were lower at 32°C leading to a reduction in chlorophyll content in the branches and an associated reduction in estimated absorbance and increase in the chlorophyll *a* specific absorption coefficient. The adjustments in the photochemical parameters led to changes in photochemical efficiencies, although these too showed that the gorgonians were coping. For example, the maximum excitation pressure, *Q*_m_, was significantly lower at 32°C than at 29.5°C. In addition, although per dry weight the amount of protein and lipids were lower at 32°C, the overall energy content in the tissues did not differ between the temperatures. Antioxidant activity either remained the same or increased following exposure to 32°C further reiterating a response that dealt with the stressor. Taken together, the capability of Caribbean gorgonian corals to modify symbiont, host and consequently holobiont parameters may partially explain their persistence on reefs faced with climate change.

## Introduction

Global climate change affects many ecosystems, including coral reefs [1]. One aspect of climate change is the rise of seawater temperatures that is anticipated to continue into the future [1, 2]. In addition, short-term fluctuations in prevailing temperatures over several hours or days are also projected to occur more frequently [3–5]. Exposure to seawater temperatures even 2°C above the mean summer maximum can adversely affect corals and their mutualistic endosymbiotic dinoflagellate algae, *Symbiodinium* spp. [3]. Numerous studies have investigated the predominantly detrimental effects of elevated seawater temperatures on scleractinian coral—*Symbiodinium* symbioses (reviewed in [6, 7, 8]), but such data on other abundant coral reef cnidarians, such as octocorals, lag behind.

In the Caribbean, for example, over the past few decades, scleractinian coral cover has dramatically declined [9, 10] concurrent with a rise in seawater temperatures by 0.2–0.4°C/decade between 1985 and 2006 [11]. On the other hand, the abundance of Caribbean octocorals, predominantly gorgonian corals, has remained the same or even increased [12–15]. In fact, gorgonian corals constitute the dominant benthic fauna on many Caribbean reefs [13, 14, 16, 17], where they provide food and shelter to a variety of invertebrates and fish [18–21]. Therefore, in order to understand the future of Caribbean reefs, it is imperative to determine the effects of potential stressors, such as elevated seawater temperatures, on gorgonian corals.

In corals, thermal stress often leads to a reduction in *Symbiodinium* numbers and/or the amount of chlorophyll within the remaining *Symbiodinium*, which is commonly referred to as coral bleaching [22]. The elevated temperatures can disrupt *Symbiodinium* photosynthesis by hindering the repair of damaged photosystems [23], increasing the production of reactive oxygen species (ROS) that impair the thylakoid membranes [24], and inhibiting enzymes responsible for carbon fixation [25]. In addition, the production of high levels of nitric oxide (NO) in thermally stressed *Symbiodinium* can result in apoptosis [26]. Sensitivity to thermal stress can vary between different *Symbiodinium* clades and sub-cladal types [27, 28].

Detrimental effects on the *Symbiodinium* may alter the nutrient exchange between the partners. *Symbiodinium* supply their host with carbohydrates, lipids, and essential and mycosporine-like amino acids [29–31], while the host provides *Symbiodinium* with carbon, nitrogen, nutrients, and an environment for photosynthesis [32–35]. Disruption of the symbiosis may alter nutrient exchange between the partners, the amount of energy required to maintain homeostasis, and drive the coral host and its symbionts to utilize their energy reserves [36]. For example, thermally stressed scleractinian corals and octocorals in the Indo-Pacific exhibit a drop in tissue reserves like lipids, proteins and carbohydrates [37–40]. In scleractinian corals, tolerance to, and the capacity to recover from, thermal stress is linked to the amounts of tissue reserves available [41, 42].

Faced with stressors, *Symbiodinium* and corals can utilize several mechanisms to mitigate dysfunction in their cells. By increasing the activities of antioxidant enzymes like superoxide dismutase (SOD) they can convert superoxide to H_2_O_2_, and then further break H_2_O_2_ down with peroxidase (POX) and catalase (CAT) to water and O_2_ [28, 43, 44]. Corals can also reduce damage to proteins by increasing the production of heat shock proteins (Hsp) [43–45]. As in *Symbiodinium*, the ability of corals to cope with thermal stress can vary between different host taxa [43, 46]. For example, *Porites cylindrica*, which possessed higher levels of SOD and Hsp than *Stylophora pistillata*, was better able to cope with, and recover from, thermal stress [43].

In contrast to the plethora of studies on the effects of elevated temperatures on scleractinian corals, only a handful of studies investigated the potential consequences of elevated seawater temperatures on Caribbean gorgonians. These studies focused only on a few parameters such as on the production of ROS, NO and Hsp90 [47, 48], the effects of pathogens on gorgonian corals at ambient and elevated temperatures [49–51] and the effects of ultraviolet radiation in conjunction with elevated temperatures [52]. We decided to employ a holistic approach to determine the effects of elevated temperature on multiple parameters of the gorgonian host, the *Symbiodinium* and the subsequent holobiont in representative species of these important Caribbean reef taxa.

## Methods

### Experimental setup

We assessed the effect of experimental short-term exposure to elevated temperature on the gorgonian species *Eunicea tourneforti*, *E*. *flexuosa*, and *Pseudoplexaura porosa*. From each species, 12 colonies located at a depth of 3–4m on a patch reef adjacent to the pier of the Instituto de Ciencas del Mar y Limnología (ICMyL), Universidad Nacional Autónoma de México (UNAM), at Puerto Morelos, México (20°52'5.23"N, 86°51'58.92"W) were sampled. Field permit was granted by Secretaria de Agricultura, Desarrollo Rural, Pesca y Alimentación (SAGARPA), permit No. GDOPA 08606.251011.3021. From each colony, two branches, 12–14cm long, were excised, attached vertically onto PVC stands, and separated into two outdoor flow-through aquaria. The temperature in both aquaria was controlled using an aquarium chiller (0.5hp Delta Star, Aqua Logic, USA) and two heaters (1000W and 1800W EasyPlug heater, Process Technologies, USA). To mimic light levels on the patch reef, garden shade cloth was placed over the tanks to reduce the incident irradiance by about 50%.

For a 14-day acclimation period, the temperature in both aquaria was held at 29.5°C. This temperature is similar to the ambient mean monthly summer (May-August) seawater temperature on local shallow reefs ranging from 29°C to 30°C [53, 54]. After the 14 acclimation days, the control aquarium was kept at the ambient 29.5°C, while the temperature in the treatment aquarium was raised, 1°C/day over three days, to 32°C. The 32°C treatment represented a 2–3°C increase above typical summer temperatures and the predicted average sea surface temperature by 2099 [55]. In addition, 32°C is 2°C greater than the bleaching threshold temperature of 30°C at this location (NOAA Coral Reef Watch Virtual Station Puerto Morelos, Mexico). Indeed, exposure to 31.5–32°C is stressful for scleractinian corals from this area and leads to coral bleaching [56–60]. When the treatment aquarium reached 32°C the experiment began. The gorgonian branches were kept in the ambient and elevated temperature treatments for five days following which the branches were processed.

### Photochemical efficiency of photosystem II

Throughout the experiment, the maximum (at dusk, Fv/Fm) and effective (at local noon, ΔF/Fm`) photochemical yields were measured daily using a Diving PAM (Walz, Germany). Plastic tubing attached to the distal end of the probe ensured a fixed distance between it and the gorgonian tissue. For each branch, the photochemical yields were measured at three locations, in the upper one-third, middle, and lower one-third of the branch. The maximum excitation pressure over photosystem II (*Q*_m_) was determined by modifying the formula in Iglesias-Prieto et al. [61] to *Q*_m_ = 1 –[(ΔF/Fm`at noon) / (Fv/Fm at dusk on the preceding day)].

### Estimated absorbance

After the five experimental days, while each gorgonian branch was immersed in seawater maintained at its respective experimental temperature (29.5°C or 32°C), the reflectance spectrum of a region located 2-4cm from the branch tip was recorded using the protocol described in Ramsby et al. [62]. Reflectance was converted to estimated absorbance (D_e_), and D_e_ at 675nm was used to calculate light absorbed by Chl *a*, and the Chl *a* specific absorption coefficient (a*_Chl a_) [63].

### Sample processing

Following absorbance determination, a 2cm fragment, 2-4cm from the branch tip, was excised, and its length and diameter were recorded. Both this fragment and the remaining branch were flash-frozen in liquid nitrogen and stored at -80°C until further processing. To isolate *Symbiodinium* cells, the 2cm fragment was ground, and the cells were separated by filtration and a series of washes with 0.2μm-filtered seawater (FSW) [64]. The *Symbiodinium* cells were re-suspended in FSW and aliquots were taken for determination of density, chlorophyll content and genetic identification.

### *Symbiodinium* cell density and chlorophyll content

From one *Symbiodinium* aliquot, in a minimum of three 100μl subsamples, *Symbiodinium* cells were counted using the FlowCAM Imaging Particle Analyzer (Fluid Imaging Technologies, Maine, USA), which pumps a liquid sample through a flow cell and captures images of the microscopic particles suspended in it [65]. *Symbiodinium* were distinguished from other particles (like cellular and sclerite debris) using a value filter based on their diameter, circle fit, and red:blue color ratio. *Symbiodinium* cell counts were standardized to surface area of the excised 2cm-long branch fragment. The *Symbiodinium* in a second aliquot were pelleted, the FSW removed, and a mixture of acetone and dimethyl sulfoxide 95:5 v/v [64] was added for 24h to extract Chlorophylls *a* (Chl *a*) and *c*_*2*_ (Chl *c*_*2*_), and their concentrations determined using the equations of Jeffrey and Humphrey [66]. The total amount of Chl *a* and *c*_*2*_ extracted was standardized to either the surface area of the excised 2cm-long fragment (Chl cm^-2^) or the number of *Symbiodinium* cells isolated from that branch fragment (Chl cell^-1^).

### Genetic identification of *Symbiodinium*

**Fig 1:**
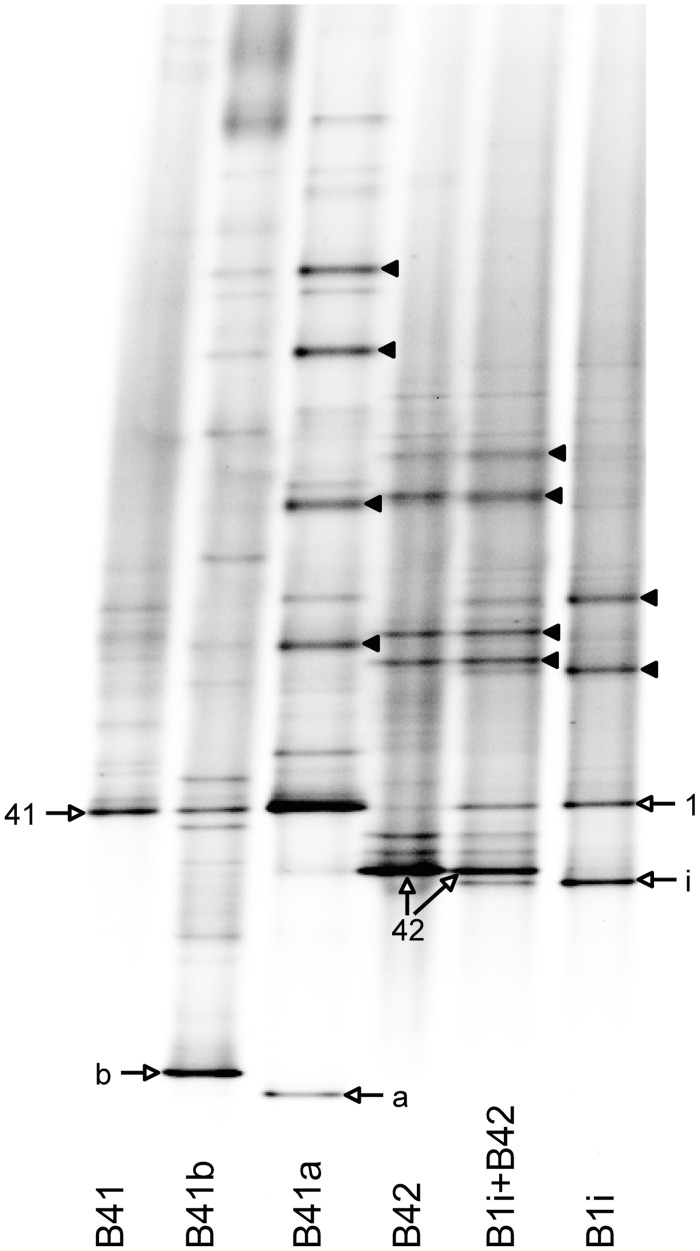
DGGE gel of *Symbiodinium* types hosted by the Caribbean gorgonian corals *Eunicea tourneforti*, *E*. *flexuosa* and *Pseudoplexaura porosa*. Bands characteristic of a particular *Symbiodinium* type are marked with white arrows. Black arrow heads denote heteroduplexes formed during the PCR process.

The *Symbiodinium* in a third aliquot were also pelleted, their DNA extracted, and the internal transcribed spacer 2 region (ITS2) of the ribosomal DNA was PCR amplified using the protocol of LaJeunesse et al. [67], with an addition of a final extension step of 30min at 72°C to reduce the formation of heteroduplexes [68]. The amplified product was separated with Denaturing Gradient Gel Electrophoresis (DGGE) on a polyacrylamide gel containing a 45–80% gradient of urea [67], with modifications described in Shirur et al. [64]. Banding profiles were compared between samples, and prominent bands from unique profiles were excised, re-amplified, and sequenced [69]. Some DGGE profiles contained multiple prominent bands, and in *E*. *flexuosa*, with the exception of the lowest band, the others were heteroduplexes (Fig 1). These bands were re-amplified [67, 68], and their constituents were separated on a polyacrylamide gel with a 55–75% denaturant gradient. The resulting bands were then excised, re-amplified, and sequenced. In addition to the ITS2, the microsatellite locus Sym15 was amplified [70], and its flanker regions were used for *Symbiodinium* lineage identification [71–74]. Both the ITS2 and Sym15 loci were sequenced at the DNA Lab at Arizona State University. DNA sequences were compared to those in GenBank, and novel sequences deposited to GenBank.

### Biochemical composition

Using the protocols described in Shirur et al. [64], from ground lyophilized gorgonian branch pieces the amount of sclerites, protein, lipid, carbohydrate and refractory content per dry weight (%g DW) and the amount of protein, lipid, carbohydrate and refractory content per organic matter (%g OM) were determined. The protein, lipid and carbohydrate content within the organic matter and their specific enthalpies of combustion [75] were then used to calculate the total energy content of tissue reserves [76].

### Enzyme activity

To quantify enzyme activity, proteins were extracted from 5cm-long frozen branch fragments using the protocol of Mydlarz and Harvell [77]. The activities of SOD, POX, and CAT in the final supernatant (crude protein extract) were measured using a Synergy HT microplate reader (Biotek, USA). To determine SOD activity, 5μl of the extract was diluted in 15ul of PBS (pH 7.8) and the activity was quantified at 37°C using the SOD assay kit (Sigma-Aldrich, USA). To measure POX activity, the protocol described in Shirur et al. [78] was modified, such that the reaction was initiated by adding 20mM H_2_O_2_ to *E*. *tourneforti* samples, and 25mM H_2_O_2_ to *E*. *flexuosa* samples. The optical density at 470nm was recorded every minute for 30min. To measure CAT activity, a 5μl aliquot of the crude extract was diluted in 150μl of 50mM H_2_O_2_ (in 50mM PBS, pH 7.0), and the breakdown of H_2_O_2_ was tracked at 240nm every 30s for 20min. Both POX and CAT assays were run at room temperature (25–27°C), and from the linear portion of the curves, their activities were calculated as the change in optical density per minute over an 8min and 10min interval, respectively. CAT activity was further converted to mM H_2_O_2_ scavenged during the assay from a standard curve generated using different concentrations of H_2_O_2_ [79–81]. Activities of all three enzymes were normalized to protein content of the aliquot used in each assay [77, 79, 82], and are reported as ΔAbs_450nm_ mg protein^-1^ and ΔAbs_470nm_ min^-1^ mg protein^-1^ for SOD and POX respectively, and mM H_2_O_2_ scavenged min^-1^ mg protein^-1^ for CAT [77, 79–85].

Protein content (mg/ml) of the extract was quantified using the RED660^™^ Protein Assay Kit (G-Biosciences). Enzyme activity in *P*. *porosa* branches could not be determined because the high mucus and lipid content interfered with the assays. Similarly, we could not obtain reliable values for CAT activity in *E*. *tourneforti*. Therefore, the activities of SOD and POX were determined for both *Eunicea* species, but CAT activity was only obtained for *E*. *flexuosa*.

### Statistical analyses

We used linear mixed effects models fit by the restricted maximum likelihood method to analyze the data. For the majority of the data, the two fixed effects in the model were the gorgonian species (*E*. *tourneforti*, *E*. *flexuosa* and *P*. *porosa*) and temperature (ambient and elevated). Data on photochemistry was analyzed with a similar mixed effects model, with the time of sampling as the third fixed effect. Since multiple colonies were sampled per species, inter-colony variation was accounted for in the mixed model by nesting each colony within its parent species, and treating it as the random effect. For some parameters, samples from one of the two branches from the same colony were not measured (e.g. sample loss), therefore data from that particular colony were excluded from the statistical analysis of that parameter.

For all data, residuals were examined for normality and homogeneity of variances, and data were square, square root, log or reciprocal transformed when these assumptions were violated (S1 Table). When significant species by temperature interactions were detected, they were explored using six non-orthogonal planned contrasts. The first three contrasts tested the effect of elevated temperature on each species separately. The other three contrasts were pairwise comparisons testing whether the magnitude of the effect of temperature differed between the three gorgonian species. For the photochemical efficiency of photosystem II parameters, ΔF/Fm`and *Q*_m_ had significant temperature by time interactions and they were explored with five orthogonal planned contrasts that tested the effect of elevated temperature on each day separately. For Fv/Fm, significant species by temperature by time interaction were explored with 27 non-orthogonal planned contrasts. The first 15 contrasts tested the effect of elevated temperature on each species on each experimental day. Then for each species, subsequent contrasts tested whether the magnitude of the effect of temperature differed between each consecutive day (day 1 versus 2, 2 versus 3, 3 versus 4, and 4 versus 5). *P*-values for all non-orthogonal planned contrasts were corrected using the method of Holm [86]. All analyses were performed using the packages “lme4” [87], “lmerTest” [88] and “multcomp” [89] with the R software version 3.0.2 [90].

## Results

### *Symbiodinium* parameters

**Fig 2:**
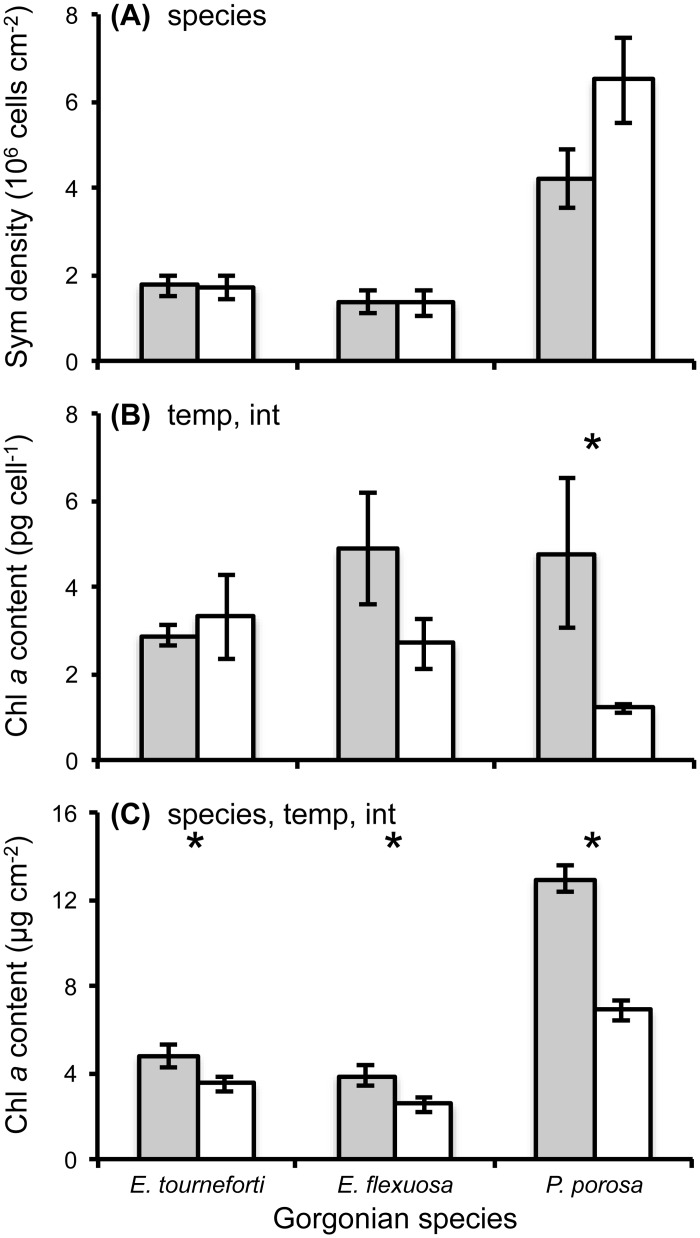
*Symbiodinium* parameters in branches of the Caribbean gorgonian corals *Eunicea tourneforti*, *E*. *flexuosa* and *Pseudoplexaura porosa* exposed to ambient and elevated temperatures. *Symbiodinium* (Sym) density (A), and chlorophyll *a* (Chl *a*) content per *Symbiodinium* cell (B), and per surface area (C) after five days at ambient, 29.5°C (gray) or elevated, 32°C (white) temperatures. Data are mean ± SE. Mixed model analyses that yielded significant species (species), temperature (temp) and/or interaction (int) effects are noted in the panels, (*) denotes comparisons in which the interaction term was significant and the planned contrast analyses detected significant temperature effects. Sample sizes in panels A, B, C were 11, 9, and 10 in *E*. *tourneforti*, 12, 11 and 11 in *E*. *flexuosa*, and 10 in A-C in *P*. *porosa*.

Exposure to five days at the elevated temperature of 32°C did not lead to a significant change in *Symbiodinium* density (Fig 2A, Tables 1 and 2, S1 Table). On the other hand, Chl *a* and *c*_*2*_ contents per *Symbiodinium* cell were significantly lower after five days at elevated temperature than their levels in branches held at ambient temperature (Fig 2B, Table 1, S1 Table). This significant difference in Chl *a* per cell was primarily driven by *P*. *porosa* since the Chl *a* content per *Symbiodinium* cell in branches of the *Eunicea* species exposed to the elevated temperature did not significantly differ from levels in branches maintained at ambient temperature (Table 2). In conjunction with no significant change in *Symbiodinium* density but a decrease in chlorophyll per *Symbiodinium* cell, the amounts of Chl *a* and *c*_*2*_ per surface area of gorgonian branches were significantly lower after five days at elevated temperature compared to their content in branches held at ambient temperature (Fig 2C, Table 1, S1 Table). The effect of temperature on Chl *a* and *c*_*2*_ content was significantly less in the *Eunicea* species than in *P*. *porosa* (Table 2). Consequently, the Chl *a*:*c*_*2*_ ratio in the *Eunicea* species did not differ between ambient and elevated temperatures while it did in *P*. *porosa* (Tables 1 and 2, S1 Table).

**Table 1:** *Symbiodinium* and holobiont parameters in branches of the Caribbean gorgonian corals *Eunicea tourneforti*, *E*. *flexuosa* and *Pseudoplexaura porosa* after exposure to ambient (29.5°C) or elevated (32°C) temperatures for five days.

Parameter	*E*. *tourneforti* (10)		*E*. *flexuosa* (11)		*P*. *porosa* (10)	
	Ambient	Elevated	Ambient	Elevated	Ambient	Elevated
*Symbiodinium* density (10^6^ cells cm^-2^)	1.73 ± 0.23 (%)	1.70 ± 0.28 (%)	1.35 ± 0.25 (^)	1.34 ± 0.30 (^)	4.22 ± 0.68	6.48 ± 1.01
Chlorophyll *a* content (pg cell^-1^)	2.88 ± 0.24 (#)	3.31 ± 0.98 (#)	4.85 ± 1.29	2.69 ± 0.59	4.77 ± 1.71	1.20 ± 0.11
Chlorophyll *c*_*2*_ content (pg cell^-1^)	0.78 ± 0.06 (#)	0.82 ± 0.21 (#)	1.55 ± 0.44	0.82 ± 0.17	1.25 ± 0.44	0.35 ± 0.03
Chlorophyll *a* content (μg cm^-2^)	4.77 ± 0.53	3.49 ± 0.37	3.84 ± 0.46	2.53 ± 0.37	12.92 ± 0.60	6.87 ± 0.47
Chlorophyll *c*_*2*_ content (μg cm^-2^)	1.30 ± 0.15	0.93 ± 0.11	1.15 ± 0.11	0.78 ± 0.11	3.43 ± 0.15	2.03 ± 0.14
Chlorophyll *a*:*c*_*2*_ ratio	3.69 ± 0.08	3.86 ± 0.13	3.29 ± 0.13	3.24 ± 0.06	3.78 ± 0.06	3.39 ± 0.09
D_e_, estimated absorbance of Chl *a*	0.44 ± 0.04 (^)	0.38 ± 0.04 (^)	0.56 ± 0.03 (^)	0.43 ± 0.04 (^)	0.74 ± 0.04	0.56 ± 0.03
a*_Chl a_ (m^2^ mg^-1^ Chl *a*)	0.02 ± 0.004	0.03 ± 0.002	0.04 ± 0.003	0.04 ± 0.003	0.01 ± 0.001	0.02 ± 0.001
SOD activity	782.91 ± 33.46 (%)	787.84 ± 43.97 (%)	485.19 ± 26.27 (^)	423.57 ± 15.55 (^)	NA	NA
POX activity	0.23 ± 0.03 (!)	0.52 ± 0.10 (!)	0.56 ± 0.15 ($)	0.36 ± 0.06 ($)	NA	NA
CAT activity	NA	NA	196.47 ± 33.86 (^)	173.61 ± 19.77 (^)	NA	NA
Sclerite content (%g DW)	84.12 ± 1.18 (^)	85.62 ± 1.40 (^)	80.88 ± 1.22 (^)	81.71 ± 1.25 (^)	50.97 ± 1.81	56.18 ± 2.31
Refractory content (%g DW)	12.13 ± 1.25	11.47 ± 1.57	13.10 ± 1.14	12.62 ± 1.38	21.09 ± 0.71	19.44 ± 1.24
Protein content (%g DW)	0.65 ± 0.06 (^)	0.48 ± 0.04 (^)	1.84 ± 0.16 (^)	1.53 ± 0.14 (^)	9.97 ± 0.56	8.37 ± 0.59
Lipid content (%g DW)	2.44 ± 0.21	2.15 ± 0.21	2.96 ± 0.25	2.71 ± 0.26	14.81 ± 0.98	12.89 ± 0.92
Carbohydrate content (%g DW)	0.95 ± 0.10 (^)	0.93 ± 0.05	1.60 ± 0.12 (^)	1.48 ± 0.07	3.16 ± 0.21	3.12 ± 0.31
Refractory content (%g OM)	73.94 ± 2.25	74.81 ± 2.32	66.33 ± 2.43	67.26 ± 3.10	43.27 ± 1.31	44.47 ± 1.59
Protein content (%g OM)	4.14 ± 0.26 (^)	3.55 ± 0.33 (^)	9.95 ± 0.89 (^)	8.75 ± 0.89 (^)	20.28 ± 0.78	19.15 ± 0.60
Lipid content (%g OM)	15.64 ± 1.44	15.33 ± 1.77	15.57 ± 1.23	15.53 ± 1.82	29.94 ± 1.07	29.30 ± 1.40
Carbohydrate content (%g OM)	6.15 ± 0.65 (^)	7.04 ± 0.65 (^)	8.65 ± 0.73 (^)	8.38 ± 0.58 (^)	6.51 ± 0.42	7.31 ± 0.78
Energy content (kJ g^-1^ OM)	8.26 ± 0.72	8.00 ± 0.80	9.94 ± 0.74	9.70 ± 0.99	17.81 ± 0.45	17.38 ± 0.58

Values are mean ± SE. (n) = number of different colonies with branches both at ambient and elevated temperatures and n = 8 (!), n = 9 (#), n = 10 ($), n = 11 (%), n = 12 (^) designating other sample sizes. a*_Chl a_ = Chlorophyll (Chl) *a* specific absorption coefficient, SOD = Superoxide dismutase (ΔAbs_450_ mg protein^-1^), CAT = Catalase (mM H_2_O_2_ scavenged min^-1^ mg protein^-1^), POX = Peroxidase (ΔAbs_470_ mg protein^-1^), DW = Dry weight, OM = Weight of organic matter, NA = Not available and pertains to assays that could not be conducted.

**Table 2:** Summary of the results of a linear mixed effects model analyses testing the effect of elevated temperature on *Symbiodinium* parameters in the Caribbean gorgonian corals *Eunicea tourneforti* (*ET)*, *E*. *flexuosa* (*EF*) and *Pseudoplexaura porosa* (*PP*).

Parameter	Species (*P*<0.05)	Temp (*P*<0.05)	Species * Temp (*P*<0.05)					
			*ET*	*EF*	*PP*	Magnitude of the effect		
		A vs E	A vs E	A vs E	A vs E	*ET* vs *EF*	*ET* vs *PP*	*EF* vs *PP*
*Symbiodinium* density (10^6^ cells cm^-2^)	(ET-EF)<PP	-						
Chlorophyll *a* content (pg cell^-1^)	-	>	-	-	>	-	<	-
Chlorophyll *c*_*2*_ content (pg cell^-1^)	-	>						
Chlorophyll *a* content (μg cm^-2^)	(ET-EF)<PP	>	>	>	>	-	<	<
Chlorophyll *c*_*2*_ content (μg cm^-2^)	(ET-EF)<PP	>	>	>	>	-	<	<
Chlorophyll *a*:*c*_*2*_ ratio	(ET-PP)>EF	-	-	-	>	-	<	-
D_e_, estimated absorbance of Chl *a*	ET<EF<PP	>						
a*_Chl a_ (m^2^ mg^-1^ Chl *a*)	EF>ET>PP	<	-	-	<	-	-	<

Comparisons where *P* < 0.05 are further delineated with ‘<‘ or ‘>‘ to denote the trend in the differences, and those where *P* > 0.05 are indicated with ‘-’. In case of a significant interaction, planned contrast analyses were run to test the effect of temperature (A = Ambient, 29.5°C, E = Elevated, 32°C) on each species, and the magnitude of the effect on each possible species pair combinations. *P* values were adjusted using the Holm’s method [86]. Chl = Chlorophyll, Temp = Temperature, a*_Chl a_ = Chl *a* specific absorption coefficient.

**Fig 3:**
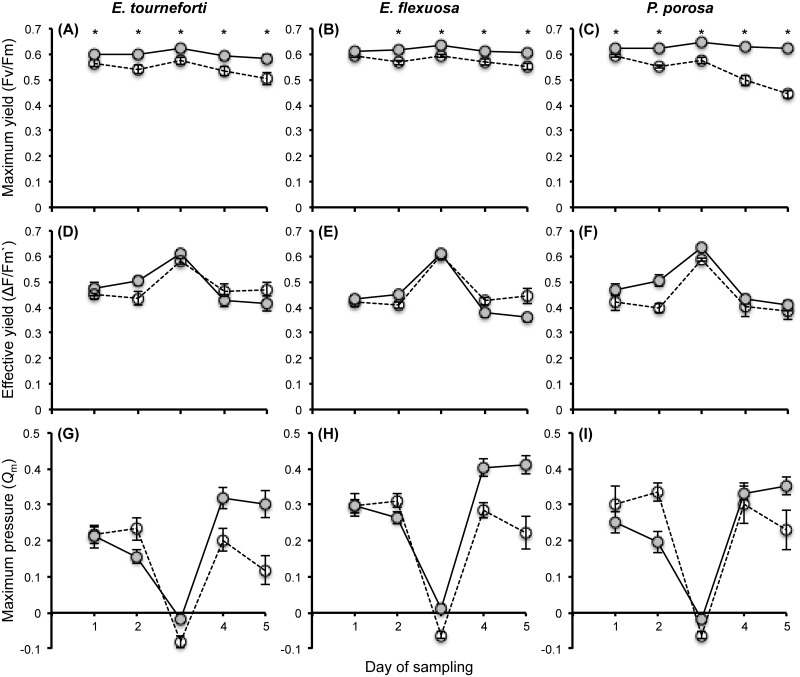
*Symbiodinium* photochemical parameters in branches of the Caribbean gorgonian corals *Eunicea tourneforti*, *E*. *flexuosa* and *Pseudoplexaura porosa* exposed to ambient and elevated temperatures. The maximum (A-C) and effective (D-F) photochemical yields, and the maximum pressure over photosystem II (G-I) of *Symbiodinium* in branches exposed to ambient, 29.5°C (solid line, gray circles) and elevated, 32°C (dashed line, open circles) temperatures for five days. Data are mean ± SE, with n = 8 for *E*. *tourneforti* and *E*. *flexuosa* and n = 6 for *P*. *porosa*. (*) indicate significant temperature effects detected in the planned contrast analyses of the three-way interaction for Fv/Fm.

Fv/Fm, ΔF/Fm`and *Q*_m_ did not significantly differ between the upper, middle and lower regions of each branch, and therefore the values from these regions were pooled. In the three gorgonian species, Fv/Fm in branches exposed to elevated temperature were significantly lower than in those maintained at ambient temperature (Fig 3A–3C, S1 Table). In *P*. *porosa*, Fv/Fm at elevated temperature declined over time, such that there was a larger reduction in Fv/Fm after four and five days than during the first three days of exposure to elevated temperature (Fig 3C). In contrast to Fv/Fm, ΔF/Fm`in the *Eunicea* species did not differ between the two temperatures, while in *P*. *porosa* a 10% reduction in ΔF/Fm`at the elevated temperature was significant compared to the control (Fig 3D–3F, S1 Table). This significant difference was probably driven by the first days at the elevated temperature. Examining ΔF/Fm`of the three gorgonian species throughout the experiment showed that on days 1, 3, and 4 there were no differences in ΔF/Fm`between branches at ambient and elevated temperatures, on day 2 there was a reduction in ΔF/Fm`at elevated temperature, but on day 5 ΔF/Fm`was actually significantly higher at the elevated compared to the ambient temperature (Fig 3D–3F, S1 Table). Mirroring the changes in photochemical efficiency, *Q*_m_ after two and three days at elevated temperature was significantly higher than the *Q*_m_ in branches held at ambient temperature (Fig 3G–3I, S1 Table). But after four and five days, *Q*_m_ in branches exposed to the elevated temperature was actually significantly lower than *Q*_m_ in branches maintained at ambient temperature (Fig 3G–3I).

Gorgonian branches exposed to elevated temperature exhibited significantly lower estimated absorbance, D_e_, and greater a*_Chl a_ compared to branches maintained at ambient temperature (Tables 1 and 2, S1 Table). The pattern in a*_Chl a_ was driven by the significant differences in *P*. porosa a*_Chl a_ since the a*_Chl a_ in branches of the *Eunicea* species did not significantly differ between branches at ambient or elevated temperatures (Table 2).

### *Symbiodinium* genotypes

Based on the ITS2 region of the ribosomal DNA, five different *Symbiodinium* types within clade B resided in the three gorgonian species (Fig 1). All 12 *E*. *tourneforti* colonies hosted a newly named *Symbiodinium* type B41 (Fig 1, Genbank accession no. KX344963). *E*. *flexuosa* also hosted new *Symbiodinium* types, with four colonies containing *Symbiodinium* type B41a (Fig 1, Genbank accession no. KX344964), and the remaining eight colonies associating with *Symbiodinium* type B41b (Fig 1, Genbank accession no. KX344965). Among *P*. *porosa* colonies, nine colonies harbored the previously characterized *Symbiodinium* type B1i (Fig 1, [72]: Genbank accession no. GU907636), one colony hosted the new *Symbiodinium*, type B42 (Fig 1, Genbank accession no. KX344981), while the remaining two colonies simultaneously associated with types B1i and B42 (Fig 1). All sequence files are available from the Genbank database (accession numbers KX344963, KX344964, KX344965, KX344981). Hosting different *Symbiodinium* types within a gorgonian species did not affect the parameters measured and therefore all branch pairs (ambient and elevated) were used in the analyses. Regardless of the *Symbiodinium* type hosted, in all three gorgonian species, the elevated temperature did not cause a change in *Symbiodinium* type.

In addition to the three gorgonian species hosting different *Symbiodinium* types, these *Symbiodinium* belonged to three different lineages. *Symbiodinium* type B41 in *E*. *tourneforti* belonged to one *Symbiodinium* lineage (Genbank accession no. KX344969). Although *E*. *flexuosa* hosted both *Symbiodinium* types B41a and B41b, the Sym15 flanker sequences, and hence the lineage, were identical for both types (Genbank accession no. KX344973). Similarly, even though *P*. *porosa* hosted *Symbiodinium* type B1i, B42, or a mixture of both, the Sym15 flanker sequences of both types were identical (Genbank accession no. KX344977). As with the ITS2 types, elevated temperature did not alter the microsatellite Sym15 flanker regions in the respective colonies and species.

### Biochemical composition of tissues

**Fig 4:**
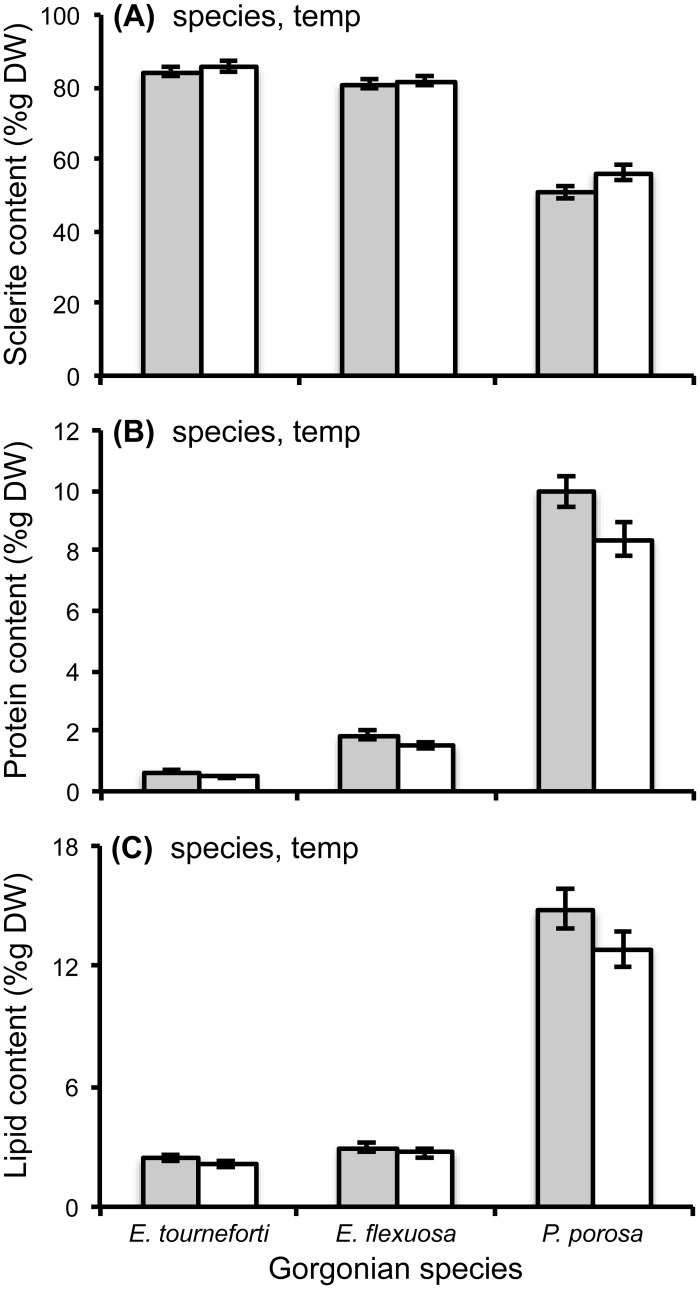
Biochemical parameters in branches of the Caribbean gorgonian corals *Eunicea tourneforti*, *E*. *flexuosa* and *Pseudoplexaura porosa* exposed to ambient and elevated temperatures. Sclerite (A), protein (B) and lipid (C) contents per dry weight (%g DW) after five days at ambient, 29.5°C (gray) and elevated, 32°C (white) temperatures. Data are mean ± SE. Mixed model analyses that yielded significant species (species) and/or temperature (temp) effects are noted in the panels. Sample sizes in panels A, B, C were 12, 12, and 10 in *E*. *tourneforti*, 12, 12 and 11 in *E*. *flexuosa*, and 10 in A-C in *P*. *porosa*.

Per dry weight (%g DW), sclerite content was significantly higher, while protein and lipid contents were significantly lower, in gorgonian branches exposed to 32°C than in those maintained at ambient temperature (Fig 4A–4C, Table 1, S1 Table). Protein content per organic matter (%g OM) was also significantly lower in gorgonian branches exposed to elevated temperature than in those held at the ambient temperature (Table 1, S1 Table). On the other hand, the carbohydrate content per dry weight and per organic matter, the lipid content per organic matter, and the total energy content of the tissues did not significantly differ between branches held at ambient and elevated temperatures (Table 1, S1 Table).

### Enzyme activity

Compared to ambient levels, exposure to 32°C for five days did not cause a significant change in the SOD activity in branches of both *Eunicea* species (Table 1, S1 Table), and CAT and POX activity in *E*. *flexuosa* branches (Table 1, S1 Table, CAT: Paired t test, *t*_11_ = 0.74, *P* = 0.475). In contrast, five days at 32°C significantly increased POX activity in branches of *E*. *tourneforti* compared to the branches held at the ambient temperature (Table 1, S1 Table).

## Discussion

Thermal fluctuations that expose coral reefs to anomalously high or low seawater temperatures for several hours or days are projected to occur more frequently in the future [3–5]. In scleractinian corals subjected to experimental conditions simulating such events, a 50–80% reduction in *Symbiodinium* density often occurs [39, 43, 91–96]. Subsequently, scleractinian corals may recover from the bleaching event. Conversely, the loss of *Symbiodinium*, compounded with the other stress responses, may lead to the demise of the host [39, 43, 94–96]. While we mimicked a short term thermal event by exposing branches of three gorgonian species, *Eunicea tourneforti*, *E*. *flexuosa*, and *Pseudoplexaura porosa*, to an elevated 32°C seawater temperature, the *Symbiodinium* densities in these branches did not significantly differ from *Symbiodinium* densities in branches from the same colonies maintained at the ambient temperature of 29.5°C. Furthermore, in *P*. *porosa*, *Symbiodinium* densities at the elevated temperature were actually higher, not lower, compared to those at the ambient temperature, although not significantly so (Fig 2). The ability to continue hosting the same *Symbiodinium* density at elevated temperatures may be one reason why Caribbean gorgonians are maintaining or increasing their abundance on Caribbean coral reefs while scleractinian coral cover is declining [9, 10, 12–15].

Although the elevated temperature did not significantly alter the *Symbiodinium* density in the three gorgonian species, the *Symbiodinium* in branches of the gorgonian corals did react to the change in environmental conditions by modifying other *Symbiodinium* parameters. For example, at 32°C there was less Chl *a* and Chl *c*_*2*_ per *Symbiodinium* cell which, in turn, affected the amount of chlorophyll per surface area (Fig 2). Absorbance was less and, concomitantly, a* was more at the elevated temperature. Chlorophyll content can be altered over short timescales in response to changes in the environment (reviewed in [97]), and is a quicker response than *Symbiodinium* re-population, following symbiont loss, which can take from six weeks [58] to over two years [98].

The adjustments in pigments and subsequent light capture could in turn affect photochemical efficiency. In scleractinian corals, thermal stress can hamper symbiont photochemistry and photosynthesis [57, 91, 92], resulting in reductions in both Fv/Fm and ΔF/Fm`[43, 99, 100], leading to *Q*_m_ values of 0.8 or above [99, 101, 102]. In the three gorgonian species, Fv/Fm at the elevated temperature was reduced, but Fv/Fm can also be lower due to activation of photoprotective processes [103]. In the *Eunicea* species, ΔF/Fm`did not mirror the reduction in Fv/Fm and was not affected by the elevated temperatures (Fig 3). In *P*. *porosa* a 10% significant reduction in ΔF/Fm`occurred, but this reduction was much smaller than the >50% reduction in ΔF/Fm`recorded in thermally stressed scleractinian corals [43, 99, 100]. Furthermore, looking at daily changes in ΔF/Fm`in all three gorgonian species demonstrated that by day 5, ΔF/Fm`was actually higher at the elevated than at the ambient temperature. Lastly, in all three gorgonian species, the *Q*_m_ in branches exposed to 32°C was either similar to or lower than the *Q*_m_ in branches held at the 29.5°C ambient temperature, with *Q*_m_ values being lower than 0.5 (Fig 3). Given the numerous parameters related to photosynthesis, in addition to the maintenance of symbiont densities, the *Symbiodinium* appeared to not be photosynthetically compromised.

Concomitantly, the elevated temperature did not lead to a change in the *Symbiodinium* genotypes in any of the three gorgonian symbioses. Lack of symbiont turnover either over time [104, 105] or following environmental perturbation or disease in gorgonian corals ([49, 78], Ramsby et al. unpubl., McCauley et al. unpubl.), other octocorals [106] and in numerous studies on scleractinian coral species [107–111] and sea anemones [27] has been demonstrated repeatedly. Although the gorgonians did not change their symbiont complement, the three gorgonian species did host different *Symbiodinium* types (Fig 1), and analysis of the microsatellite Sym15 flanker region indicated that these *Symbiodinium* belonged to three distinct lineages of the “B1” radiation [72, 112]. *Symbiodinium* type B41 in *E*. *tourneforti* in our study (previously referred to as B1l in [64]) fell within the same *Symbiodinium* lineage as the *Symbiodinium* hosted by *E*. *flexuosa* at >20m depth [73] and *Symbiodinium endomacracis* that associate with the scleractinian coral *Madracis* sp. [74] in the Caribbean. *Symbiodinium* types B41a and B41b (previously referred to as B1b in [64]) which we found in *E*. *flexuosa*, belong to a separate lineage that includes the *Symbiodinium* inhabiting *E*. *flexuosa* found at <5m depth [73]. *Symbiodinium* types B1i and B42 found in *P*. *porosa* belong to a novel lineage. Given the lack of a change in *Symbiodinium*, any response and potential acclimation to the stressor was accomplished by modifying parameters within the existing host/symbiont genotypic combination. The gorgonian species hosting different *Symbiodinium*, with these symbionts exhibiting different physiologies [62], may have contributed to the differences between the response of the gorgonian species to the elevated temperature.

In addition to elevated temperature potentially affecting *Symbiodinium*, the entire symbiosis, including the host may be detrimentally affected [113]. Activation of cellular mechanisms to deal with stressors could increase the amount of energy required to maintain homeostasis [45, 114], and thereby alter metabolism, and consequently the biochemical composition of tissues. In our study, compared to branches maintained at the ambient temperature, gorgonian branches exposed to elevated temperature exhibited higher sclerite content driven by lower protein and lipid contents per dry weight (Fig 4), and also lower protein content when protein was assessed per organic matter. Thus, as seen in scleractinian corals [39, 115], exposure to elevated temperature led to a reduction in the amount of tissue biomass present within gorgonian branches. Compared to scleractinian corals, however, this reduction was relatively small. For example, in bleached scleractinian corals, total biomass, mean protein, lipid and carbohydrate contents can be 40–70% lower [98, 116], and mean energy content can be 22–37% lower [115] compared to tissues of unbleached corals. In our study, in the branches of the *Eunicea* species and *P*. *porosa*, protein per organic matter at the elevated temperature was only 6.5–14.3% lower compared to branches at ambient temperature. Furthermore, lipid, carbohydrate, and total energy content in tissues did not significantly differ between the gorgonian branches at ambient and elevated temperatures. Thus, despite some changes in biomass and protein content, exposure to elevated temperature did not affect the amount of energy available to these gorgonian species.

An integral part of maintaining the symbiosis under thermal stress involves managing the levels of ROS produced in the chloroplasts of *Symbiodinium* and the mitochondria of the host [26, 44, 117]. Oxidative outbursts after exposure to elevated temperature have been recorded in Caribbean gorgonian corals, and their magnitude can vary between species [47]. Both *Symbiodinium* and their host cnidarian possess antioxidant enzymes that neutralize ROS [26, 28, 43, 44]. In this study, SOD activity did not significantly vary between branches of the *Eunicea* species at ambient and elevated temperatures. Therefore, despite the nearly two-fold difference in SOD activity between the *Eunicea* species, basal levels of SOD in both species were sufficient to convert any excess O_2_^-^ to H_2_O_2_ [44, 45]. H_2_O_2_ itself is damaging because it can readily diffuse across membranes from one partner to the other, affect distant cell organelles, and trigger apoptosis [26, 44]. The enzymes POX and CAT neutralize H_2_O_2_. POX activity in *E*. *tourneforti* was two times higher in branches exposed to elevated temperature than in those maintained at ambient temperature while in *E*. *flexuosa* branches, POX and CAT activity did not differ between the two temperatures. Therefore, the *Eunicea* species maintained or increased the activities of antioxidant enzymes when exposed to elevated temperature indicating that they managed oxidative stress.

Looking at *Symbiodinium*, holobiont and enzymatic parameters, the three gorgonian-*Symbiodinium* symbioses examined dealt with the potential stress of elevated temperature, although the way in which they did so differed. In *P*. *porosa* many *Symbiodinium* parameters were modified in response to the elevated temperature. Not only did the largest reduction in Fv/Fm occur in *P*. *porosa* but Fv/Fm also progressively declined with the duration of exposure to elevated temperature. A reduction in chlorophyll content of symbiont cells also only occurred in *P*. *porosa*. Among the three species *P*. *porosa* has the highest symbiont density and pigment content in tissues, and the lowest a*_Chl a_ (this study, [62, 64]). These parameters along with attributes of the photosynthesis-irradiance curves, led Ramsby et al. [62] to hypothesize that the symbionts in *P*. *porosa* were comparatively less efficient at absorbing and utilizing light than those in *E*. *tourneforti*. Since high light levels can exacerbate thermal stress, the inefficient utilization of light may alleviate the negative effects of elevated temperature, and promote photoacclimation through adjusting photochemistry over losing symbiont cells from tissues. Furthermore, net photosynthesis in *P*. *porosa* is two to three times higher than in *E*. *tourneforti* [62], and *P*. *porosa* possess significantly greater amounts of tissue reserves than the *Eunicea* species (this study, [64]). Thus, the low efficiency of photosynthesis per symbiont cell coupled with high net photosynthesis and tissue resources may enable *P*. *porosa* to tolerate disruptions in symbiont photosynthesis that may occur when exposed to elevated temperature.

In the *Eunicea* species, the modifications that occurred in the symbioses were predominantly at the holobiont rather than the symbiont level. Exposure to elevated temperature led to greater reductions in mean protein content per organic matter in tissues of *E*. *tourneforti* (14.30%) and *E*. *flexuosa* (12.11%) than in those of *P*. *porosa* (6.51%) and to a doubling of POX activity in *E*. *tourneforti*. Even under ambient conditions, the *Eunicea* species had lower *Symbiodinium* density, pigment content, and energy reserves than *P*. *porosa* (this study, [64]). Due to the lower tissue resources at their disposal, the *Eunicea* species may attenuate changes in symbiont parameters by maintaining or increasing antioxidant activity to survive unfavorable conditions.

In the literature, bleaching of Caribbean gorgonian corals is seldom reported [118–121] and the three gorgonian species in our study did not exhibit a decline in *Symbiodinium* density although a reduction in the amount of chlorophyll per gorgonian surface area did occur, with *P*. *porosa* having a larger drop than within the *Eunicea* species. The varied responses of the gorgonian corals in our study match the inter-species differences in a visual assessment of bleaching on the reef [121]. In a 2005 bleaching event in Puerto Rico, 22% of *Pseudoplexaura* spp. colonies bleached [121]. In contrast, bleaching was observed in only a few *E*. *flexuosa* colonies and none of the other *Eunicea* species bleached [121]. Our study, however, suggests that even with a reduction in chlorophyll at 32°C, *Symbiodinium* photosynthesis in *P*. *porosa* was not compromised, and therefore the changes in pigment content were potentially part of an acclimatory response. This may explain why, with the exception of *Muricea* sp., the gorgonian species that were observed bleached in 2005 survived the thermal event [121]. Furthermore, in the 2005 bleaching event, bleaching in the Caribbean gorgonian species occurred much after most scleractinian corals, hydrocorals and zoanthids had bleached [121]. Taken together, the response of the gorgonian symbioses to elevated temperature in this study, and the few reports on bleaching in gorgonian corals [118–121], suggest that in the Caribbean, gorgonian corals may be comparatively more tolerant to thermal stress than many scleractinian coral species.

## Supporting information

S1 Table Results of the mixed effects model analyses testing the effect of elevated temperature (32°C) on *Symbiodinium* (Sym) and holobiont parameters in the Caribbean gorgonian corals *Eunicea tourneforti*, *E*. *flexuosa* and *Pseudoplexaura porosa*. (DOCX)
