# Supplementary material for: The effects of elevated seawater temperatures on Caribbean gorgonian corals and their algal symbionts, Symbiodinium spp
Source: PLoS One. 2017 Feb 2;12(2):e0171032. doi: 10.1371/journal.pone.0171032 (PMC5289496; doi:10.1371/journal.pone.0171032)
Supplement: S1 Table — (DOCX) [file pone.0171032.s001.docx]

# Supporting Information

**S1 Table.** **Results of the mixed effects model analyses testing the effect of elevated temperature (32°C) on *Symbiodinium* (Sym) and holobiont parameters in the Caribbean gorgonian corals *Eunicea tourneforti*, *E. flexuosa* and *Pseudoplexaura porosa*.**

| **Parameter** | **Factor** | ***MS*** | ***F*** | ***P*** |
| --- | --- | --- | --- | --- |
| Sym density**^#^** (10^6^ cells cm^-2^**)** | Species | 6.35 | *F_2,20_* = 35.43 | **<0.001** |
|  | Temperature | 0.37 | *F_1,30_* = 2.07 | 0.1161 |
|  | Species*Temperature | 0.49 | *F_2,30_* = 2.73 | 0.081 |
| Chl *a* content^$^ (pg cell^-1^) | Species | 0.29 | *F_2,31.4_* = 1.10 | 0.227 |
|  | Temperature | 2.58 | *F_1,31.4_* = 11.76 | **<0.001** |
|  | Species*Temperature | 0.80 | *F_2,31.4_* = 3.43 | **0.025** |
| Ch *c_2_* content^$^ (pg cell^-1^) | Species | 0.17 | *F_2,31.4_* = 1.91 | 0.165 |
|  | Temperature | 0.85 | *F_1,31.4_* = 9.51 | **0.004** |
|  | Species*Temperature | 0.22 | *F_2,31.4_* = 2.38 | 0.109 |
| Chl *a* content (μg cm^-2^) | Species | 119.76 | *F_2,28_* = 85.60 | **<0.001** |
|  | Temperature | 128.38 | *F_1,28_* = 91.75 | **<0.001** |
|  | Species*Temperature | 38.35 | *F_2,28_* = 27.41 | **<0.001** |
| Chl *c_2_* content (μg *a* cm^-2^) | Species | 10.64 | *F_2,28_* = 86.37 | **<0.001** |
|  | Temperature | 7.84 | *F_1,28_* = 63.61 | **<0.001** |
|  | Species*Temperature | 1.80 | *F_2,28_* = 14.57 | **<0.001** |
| Chl *a*:*c_2_* ratio | Species | 0.80 | *F_2,28_* = 11.41 | **<0.001** |
|  | Temperature | 0.12 | *F_1,28_* = 1.78 | 0.192 |
|  | Species*Temperature | 0.38 | *F_2,28_* = 5.43 | **0.010** |
| D_e_ | Species | 0.41 | *F_2,31_* = 17.92 | **<0.001** |
|  | Temperature | 0.26 | *F_1,31_* = 22.71 | **<0.001** |
|  | Species*Temperature | 0.04 | *F_2,31_* = 1.64 | 0.210 |
| *a***_Chl a_*^†^ (m^2^ mg^-1^ Chl *a*) | Species | 6103.5 | *F_2,28_* = 45.68 | **<0.001** |
|  | Temperature | 2378.1 | *F_1,28_* = 17.80 | **<0.001** |
|  | Species*Temperature | 579.1 | *F_2,28_* = 4.33 | **0.023** |
| Maximum yield (Fv/Fm) | Species | 0.0002 | *F_2,19_* = 1.46 | 0.257 |
|  | Temperature | 0.03 | *F_1,19_* = 185.08 | **<0.001** |
|  | Time | 0.02 | *F_4,76_* = 110.79 | **<0.001** |
|  | Species*Temperature | 0.002 | *F_2,19_* = 11.42 | **<0.001** |
|  | Species*Time | 0.001 | *F_8,76_* = 8.86 | **<0.001** |
|  | Temperature*Time | 0.009 | *F_4,76_* = 56.78 | **<0.001** |
|  | Species*Temperature*Time | 0.002 | *F_8,76_* = 14.72 | **<0.001** |
| Effective yield (ΔF/Fm`) | Species | 0.0007 | *F_2,19_* = 1.46 | 0.404 |
|  | Temperature | 0.002 | *F_1,19_* = 185.08 | 0.132 |
|  | Time | 0.19 | *F_4,75.7_* = 110.79 | **<0.001** |
|  | Species*Temperature | 0.004 | *F_2,19_* = 11.42 | **0.021** |
|  | Species*Time | 0.002 | *F_8,75.6_* = 8.86 | **0.034** |
|  | Temperature*Time | 0.02 | *F_4,75.6_* = 56.78 | **<0.001** |
|  | Species*Temperature*Time | 0.0008 | *F_8,75.6_* = 14.72 | 0.523 |
| Maximum pressure (*Q*_m_) | Species | 0.01 | *F_2,19_* = 5.11 | **0.017** |
|  | Temperature | 0.04 | *F_1,19_* = 14.01 | **0.001** |
|  | Time | 0.54 | *F_4,75.8_* = 188.22 | **<0.001** |
|  | Species*Temperature | 0.009 | *F_2,19_* = 3.12 | 0.067 |
|  | Species*Time | 0.003 | *F_8,75.8_* = 1.03 | 0.424 |
|  | Temperature*Time | 0.104 | *F_4,75.6_*= 36.42 | **<0.001** |
|  | Species*Temperature*Time | 0.0009 | *F_8,75.6_* = 0.31 | 0.960 |
| SOD activity^#^ | Species | 279.38 | *F_1,21_* = 93.85 | **<0.001** |
|  | Temperature | 5.30 | *F_1,21_* = 1.78 | 0.196 |
|  | Species*Temperature | 5.86 | *F_1,21_* = 1.97 | 0.175 |
| POX activity^#^ | Species | 0.01 | *F_1,16_* = 0.49 | 0.493 |
|  | Temperature | 0.02 | *F_1,16_* = 0.93 | 0.349 |
|  | Species*Temperature | 0.26 | *F_1,16_* = 9.80 | **0.006** |
| Sclerite content^œ^ (%g DW) | Species | 17410897 | *F_2,31_* = 127.05 | **<0.001** |
|  | Temperature | 1778126 | *F_1,31_* = 12.98 | **0.001** |
|  | Species*Temperature | 276442 | *F_2,31_* = 2.02 | 0.150 |
| Refractory content (%g DW) | Species | 79.28 | *F_2,27_* = 15.28 | **<0.001** |
|  | Temperature | 13.86 | *F_1,27_* = 2.67 | 0.114 |
|  | Species*Temperature | 2.30 | *F_2,27_* = 0.44 | 0.647 |
| Protein content^$^ (%g DW) | Species | 4.06 | *F_2,31_* = 433.07 | **<0.001** |
|  | Temperature | 0.28 | *F_1,31_* = 30.32 | **<0.001** |
|  | Species*Temperature | 0.006 | *F_2,31_* = 0.59 | 0.560 |
| Lipid content^#^ (%g DW) | Species | 5.20 | *F_2,28_* = 189.84 | **<0.001** |
|  | Temperature | 0.33 | *F_1,28_* = 12.04 | **0.002** |
|  | Species*Temperature | 0.05 | *F_2,28_* = 1.92 | 0.166 |
| Carb content^#^ (%g DW) | Species | 2.39 | *F_2,31_* = 98.25 | **<0.001** |
|  | Temperature | 0.008 | *F_1,31_* = 0.33 | 0.571 |
|  | Species*Temperature | 0.003 | *F_2,31_* = 0.12 | 0.885 |
| Refractory content (%g OM) | Species | 932.52 | *F_2,27_* = 54.35 | **<0.001** |
|  | Temperature | 11.12 | *F_1,27_* = 0.648 | 0.428 |
|  | Species*Temperature | 0.02 | *F_2,27_* = 0.001 | 0.999 |
| Protein content^#^ (%g OM) | Species | 7.46 | *F_2,31_* = 150.05 | **<0.001** |
|  | Temperature | 0.51 | *F_1,31_* = 10.35 | **<0.001** |
|  | Species*Temperature | 0.006 | *F_2,31_* = 0.11 | 0.895 |
| Lipid content (%g OM) | Species | 305.25 | *F_2,28_* = 36.19 | **<0.001** |
|  | Temperature | 1.67 | *F_1,28_* = 0.20 | 0.660 |
|  | Species*Temperature | 0.48 | *F_2,28_* = 0.10 | 0.945 |
| Carb content (%g OM) | Species | 10.13 | *F_2,31_* = 3.69 | **0.036** |
|  | Temperature | 3.73 | *F_1,31_* = 1.36 | 0.253 |
|  | Species*Temperature | 2.45 | *F_2,31_* = 0.89 | 0.419 |
| Energy content (kJ g^-1^ OM) | Species | 99.12 | *F_2,28_* = 53.37 | **<0.001** |
|  | Temperature | 1.51 | *F_1,28_* = 0.81 | 0.375 |
|  | Species*Temperature | 0.06 | *F_2,28_* = 0.03 | 0.967 |

For most parameters, gorgonian species and temperature were the two fixed effects in the model. In the antioxidant activity analysis, the fixed effect species had only two levels because SOD and POX activities were only measured in the *Eunicea* species. Models used to analyze the photochemical parameters had an additional fixed effect: time (days 1-5). In all models, parent colony nested within its respective species was the random effect. *MS* = mean square error, *F_μ,ν_* = *F*-ratio and degrees of freedom, *P* = probability of the null, <0.05 in bold. To satisfy the assumptions of normality and homoscedasticity, some data were (#) square root, ($) log, (†) reciprocal, or (œ) square transformed. Chl = Chlorophyll, D_e_ = Estimated absorbance of Chl *a*, *a***_Chl a_* = Chl *a* specific absorption coefficient, SOD = Superoxide dismutase (ΔAbs_450_ mg protein^-1^), POX = Peroxidase (ΔAbs_470_ mg protein^-1^), Carb = Carbohydrate, DW = Dry weight, OM = Weight of organic matter.
